# Factors associated with falls in patients with knee osteoarthritis: A cross-sectional study

**DOI:** 10.1097/MD.0000000000032146

**Published:** 2022-12-02

**Authors:** Rakhmad Rosadi, Amornthaep Jankaew, Po-Ting Wu, Li-Chieh Kuo, Cheng-Feng Lin

**Affiliations:** a Institute of Allied Health Sciences, College of Medicine, National Cheng Kung University, Tainan, Taiwan; b Department of Physiotherapy, University of Muhammadiyah Malang, Malang, Indonesia; c Department of Orthopedics Surgery, National Cheng Kung University Hospital, Tainan, Taiwan; d Department of Occupational Therapy, College of Medicine, National Cheng Kung University, Tainan, Taiwan; e Department of Physical Therapy, College of Medicine, National Cheng Kung University, Tainan, Taiwan; f Physical Therapy Center, National Cheng Kung University Hospital, Tainan, Taiwan.

**Keywords:** elderly patients, knee osteoarthritis, proprioception, range of motion, risk of falls

## Abstract

Falls represent an important adverse effect associated with knee osteoarthritis and result in a significant financial burden on the healthcare system. Therefore, identification of fall predictors is essential to minimize fall incidence. However, few studies have investigated falls and fall predictors, particularly focused on the fear of falls and proprioception. In this study, we investigated significant fall predictors in patients with knee osteoarthritis in Malang, Indonesia. Our findings may serve as useful guidelines to develop geriatric fall prevention programs. This cross-sectional survey using purposive sampling was performed between April and July 2021 and included 372 participants. We recorded the following data: sociodemographic and medical history questionnaire responses, visual analog scale scores, Hopkins falls grading scale scores, Fall Efficacy Scale-International scores, proprioception test findings, knee injury and osteoarthritis outcome score (KOOS), range of motion (ROM), chair stand test and the timed up and go test performance. Data were analyzed using the chi-square and *t* tests, and multivariate logistic regression to determine significant fall predictors. Multivariate logistic regression analysis showed a lower risk of falls in patients with better proprioception and ROM than in the other groups (odds ratio 0.55 vs 0.96). The risk of falls was higher in patients with higher KOOS symptoms, fear of falls, diagnosis of low back pain and diabetes mellitus, and increased body mass index than in the other groups (odds ratio 1.41, 2.65, 1.27, 3.45, and 1.10, respectively. Our study shows that knee proprioception and ROM serve as protective factors against falls, whereas KOOS symptoms, fear of falls, low back pain, diabetes mellitus, and body mass index were associated with a high risk of falls, with diabetes mellitus and fear of falls being the most significant risk factors. These findings may be useful to policy makers to develop a fall prevention program that can be implemented in community health care centers across Indonesia to deliver individualized, person-centered care and improve fall prevention strategies through a systematic process comprising evaluation, intervention, and monitoring to minimize fall risk.

## 1. Introduction

Falls constitute a leading cause of disability and morbidity among elderly individuals, and fall-induced complications result in a significant financial burden to the healthcare system.^[1,2]^ Compared with other Southeast Asian countries, the risk of falls is higher in the institutionalized elderly population of Indonesia. Among these individuals, up to 42.8% were reported to have a severe incidence.^[3]^ Previous research^[4,5]^ has shown a 2-fold increase in fall prevalence among the community-dwelling Indonesian elderly population (aged ≥ 50 years) over the last 2 years owing to lack of awareness regarding falls and fall risk. Therefore, identification of factors associated with fall risk is necessary to minimize fall incidence in this population.

Previous research has shown that patients with symptomatic knee osteoarthritis (OA) experience joint pain and stiffness, which lead to functional decline, and owing to these physical limitations, the fall risk is higher in this population than in healthy older adults without knee discomfort or OA symptoms.^[6]^ A study performed in 2017 observed that nearly 50% of patients with knee OA experienced falls.^[7,8]^ The high risk of falls in patients with knee OA is attributable to several factors such as knee instability, muscle weakness, and significant decline in basic functional ability.^[9,10]^ In addition to deterioration of functional ability, personal factors may predispose these individuals to falls, 1 of these being the fear of falls, which may be attributable to the fact that these individuals have known fall hazards, including gait impairment and variability, decreased static postural control, muscle weakness, pain, impaired proprioception, and obesity. Clinical tests, such as the timed up and go test (TUG)^[11]^ and those that evaluate functional balance and walking gait^[12]^ or muscle strength^[13]^ are used to predict fall risk in the general population. Although these clinical tests are associated with fall events in the general population, the results of these tests may be more reliable as predictors in patients with knee OA, who frequently show mobility and strength limitations. Individuals who fear falls may lose confidence in their ability to perform typical tasks without falling and lose their independence and limit their social connections, which can lead to cognitive impairment and consequently, a higher risk of falls. Therefore, it is important to urgently establish effective fall prevention programs for elderly patients with knee OA. However, fall prevention programs that include fall and fall risk assessments to facilitate prompt intervention are currently unavailable in Indonesia. This study provides epidemiological data regarding falls and fall risk, which can be useful to support the development of fall prevention programs in Indonesia. A systematic review has reported fall predictors such as impaired balance, muscle weakness, comorbidities, and symptomatic joint difficulties in patients with knee OA; however, the effects of the fear of falls and proprioception on fall incidence in patients with OA remain unknown.^[9]^ In this study, we investigated predictors of fall incidence (patient characteristics, medical history, knee function, fear of falls, knee range of motion (ROM), proprioception, muscle strength, and dynamic balance) in patients with knee OA in Malang City, Indonesia. We hypothesized that individuals with a history of falls within 6 months preceding study enrollment were more likely to have poorer proprioception, knee function, and fear of falls than those without a fall history.

## 2. Methods

### 2.1. Study design and participants

This cross-sectional study included patients recruited from Puskesmas Rampal Celaket and Posyandu Lansia Samaan, senior community service centers in Malang, Indonesia between April and July 2021. Participants (the study included both sexes) were recruited using a purposive sampling technique, using the following inclusion criteria: diagnosis of unilateral or bilateral knee OA by orthopedic surgeons, Kellgren-Lawrence grades 1 and 2 (mild-to-moderate knee OA), age > 50 years, and referral to health services or rehabilitation services for < 1 year. Exclusion criteria were as follows: lack of fluency in the Indonesian language, a history of knee surgery, nervous system disorders, a history of periarticular fractures and rheumatoid arthritis. Sample size was calculated using the online sample size calculator with 95% confidence intervals (CI), 5% margin of error, and 50% population proportion; therefore, finally 372 participants were recruited for this study.

### 2.2. Ethical considerations

This study was approved by the Institutional Review Board of Muhammadiyah University Malang’s Health Research Ethics Committee (No. E.5.a/160.KEPK-UMM/VII/2020). Participants were explained all procedures and signed the consent form prior to data collection. Informed consent was also obtained from patients for the purpose of publication of this manuscript.

### 2.3. Outcome measurements

#### 2.3.1. Sociodemographic characteristics, medical history, and pain.

Patient interviews were performed and the following information was recorded: age, sex, body mass index (BMI), lesion laterality (unilateral/bilateral knee involvement), a history of sleeping pill consumption, and comorbidities such as pneumonia, diabetes, neurological disorders, heart disease, and low back pain.

Knee pain experienced during functional activity was measured using a visual analog scale, which is a visual image that corresponds to the level of pain experienced by patients; the visual analog scale is a 10 cm line divided into 1-cm notches to which patients assign a numeral value to rate their pain, with grade 0 = no pain and grade 10 = the most severe pain.^[14]^

#### 2.3.2. Fall history.

The Hopkins falls grading scale was used to determine the fall history within 6 months prior to study enrollment.^[15]^ This study used a 4-point scale (Grades 1–4) to assess fall severity as follows: A near-fall (Grade 1) is distinguished from a fall for which an individual did not receive medical attention (Grade 2), a fall associated with medical attention but not hospitalization (Grade 3), and a fall associated with hospitalization (Grade 4). Based on this questionnaire and fall incidence, patients were categorized into the fall and non-fall groups. This measurement has shown good inter-rater reliability when applied to patients with knee OA.^[13]^

#### 2.3.3. Fear of falls.

Fear of falls was assessed using the falls efficacy scale-international questionnaire,^[16,17]^ which includes 16 questions, each of which is assigned a score ranging from 1 to 4. Score 1 = no fear of falls and score 4 = significant fear of falls. The Indonesian version of the falls efficacy scale-international questionnaire with high reliability and validity was used in this study.^[18]^

#### 2.3.4. Knee injury and osteoarthritis outcome score questionnaire.

The knee injury and osteoarthritis outcome score (KOOS) questionnaire was used to assess activities of daily living (ADL). The 42-question KOOS tool includes the following 5 subscales: 7 questions regarding other symptoms, 9 questions regarding pain, 17 questions regarding ADLs, 5 questions regarding sport and recreational activities, and 4 questions regarding quality of life. Each question was scored on a scale of 0 to 4, with 0 = no difficulty and 4 = significant knee difficulties. The total score on each subscale is calculated by adding all scores and dividing this value by the mean; high scores indicate no difficulties with functional ability, and low scores indicate severe functional disability. We used the Indonesian version of the KOOS, with predetermined validity and reliability (intraclass correlation coefficient = 0.97) for evaluation of knee OA in the Indonesian population.^[19,20]^

#### 2.3.5. Range of motion.

Knee ROM was measured using a universal goniometer, an instrument that measures the angle or degree in a human joint with a fulcrum on the bone. Knee ROM was measured in the supine and prone positions for knee extension and flexion, respectively. The goniometer was placed at the center of movement (lateral epicondylus), and patients were instructed to perform maximum knee flexion and extension movements. The goniometer was thereafter moved to follow the direction of motion from the beginning to the end, and the reading indicated by the arrow on the goniometer was recorded.^[5]^ We performed 3 repetitions in each direction without any rest between repetitions, and the mean value was recorded.

#### 2.3.6. Evaluation of proprioception.

A lower limb-matching task was used to evaluate proprioception in seated and blinded patients. Each patient’s unaffected leg was raised to a random height, and he/she was instructed to maintain this position while the affected leg was raised thrice to match the position of the unaffected leg. The mean measurement error for each leg was calculated using a protractor inscribed on a vertical transparent acrylic sheet (60 cm × 60 cm × 1 cm) positioned between the legs and marked in 2° increments.^[21]^

#### 2.3.7. The chair stand test.

The chair stand test (CST), which utilizes the 30-s CST ^[22]^ is a valid and reliable tool to test muscle strength. The 30-s CST records the number of times an individual stands up from a chair and sits down within 30 s. A normal chair (seat height 40 cm) with armrests and no backrest was used. Patients were initially seated on a chair with their backs straight and were instructed to look straight ahead and rise at their own pace with their arms folded over their chest after the “1, 2, 3, go” signal. The same chair and settings were used for all patients. The CST duration was counted when the patient started to stand up and was completed when he/she sat on the chair.

#### 2.3.8. The timed up and go test.

The TUG test used to assess dynamic balance was performed as follows: Patients were instructed to sit on a chair with a seat height of 46 cm; They were instructed to stand up and walk to a 3 m cone at a specified location; Patients were instructed to turn around, walk back, and sit on the chair. The time interval between standing up erect and being seated on the chair was recorded. Those who required > 13.5 s for the TUG test were considered to have poor dynamic balance, whereas those who required less time for this test were considered to have good dynamic balance.^[23]^

### 2.4. Statistical analysis

Univariate descriptive statistics (frequencies and percentages) were used for categorical variables and means and standard deviations for continuous variables. The association between variables was determined using an independent *t* test or the chi-square test depending on the variable. Univariate and multivariate binary logistic regression analyses were used to analyze the correlations between the main outcome variable (falls) and fall predictors (stepwise backward elimination). The stepwise backward elimination method was used to determine the variables most strongly associated with the outcome to obtain the best model. Factors that showed a statistically insignificant association with the outcome were eliminated using a stepwise backward elimination process. This method has previously been used for similar analyses^[4]^; therefore, predictors that were statistically significant on univariate analysis (*P* value < 0.20) were subjected to multivariate analyses. The SPSS software, version 20 (SPSS, Chicago, IL) was used for all analyses.

## 3. Results

The study included 372 patients with OA (mean age 68.02 ± 5.01 years). Most patients 231 (62.1%) were diagnosed with unilateral knee OA, and 112 (30.1%) patients reported a history of falls within 6 months prior to study recruitment (mean falls 1.34 ± 0.98). All patients reported a history of sleeping pill use over 6 months prior to study enrollment (Table 1).

**Table 1:** Basic Characteristics of Study Sample (N = 372).

Variables	N	%	M ± SD
***Patient Characteristics***			
**Age (yrs**)			68.02 ± 5.01
**Sex**			
Male	51	13.7	
Female	321	86.3	
**Body Mass Index (BMI**)			25.48 ± 3.88
**Legs Affected**			
Unilateral	231	62.1	
Bilateral	141	37.9	
***Falls History within 6 months***			
**Falls**			
Non Fallers	260	69.9	
Fallers	112	30.1	
**Frequency of falls**			1.34 ± 0.98
No Fall	260	69.9	
1	63	16.9	
2	10	2.7	
3	20	5.4	
4	10	2.7	
>4	9	2.4	
**Grade of Falls**			
Grade 1	10	8.93	
Grade 2	80	71.43	
Grade 3	21	18.75	
Grade 4	1	0.89	
***Medical History***			
**Sleeping Pill**			
Yes	372	100	
No	0	0	
**Pneumonia**			
No	363	97.6	
Yes	9	2.4	
**Diabetes**			
No	324	87.1	
Yes	48	12.9	
**Neurology Problem (parkinson, stroke**)			
No	362	97.3	
Yes	10	2.7	
**Heart Disease**			
No	240	64.5	
Yes	132	35.5	
**Low Back Pain**			
No	199	53.5	
Yes	173	46.5	
***Knee Functionality***			
**Fear of Falling**			23.22 ± 6.78
**KOOS**			
Symptom			17.74 ± 3.27
Pain			15.62 ± 4.81
Function in activity daily living (ADL)			26.11 ± 8.15
Function in Sport and Recreation			10.65 ± 5.58
knee-related Quality of Life			8.90 ± 2.70
**ROM Knee(degree**)			123.19 ± 16.38
**Timed Up Go Test (TUG) (second**)			13.78 ± 4.41
**Proprioception Test (degree**)			5.73 ± 3.69
**Knee Pain (VAS**)			3.14 ± 2.33
**Chair Stand Test**			9.02 ± 3.79

BMI = body mass index, KOOS = knee injury and osteoarthritis outcome source, M = mean, ROM = range of motion, SD = standard deviation, TUG = timed up and go test, VAS = visual analogue scale.

Age, BMI, diabetes mellitus, and neurological disorders were significantly different between the fall and non-fall groups (Table 2). The BMI (26.60 kg/m^2^ ± 3.98 kg/m^2^) and prevalence of diabetes mellitus (60.4%) and neurological disorders was higher in patients in the fall versus non-fall group. We observed significant differences in the fear of falls, all KOOS subscales, ROM, TUG, proprioception, and knee pain among all indicators of knee function (*P* < .001 for all variables). Scores for fear of falls (30.19 ± 7.93), TUG (16.22 ± 6.07), proprioception tests (7.25 ± 3.52), and knee pain (4.62 ± 2.25) were higher in the fall group. All KOOS subscales (16.85 ± 3.21, 14.14 ± 3.97, 24.17 ± 7.49, 9.51 ± 5.01, and 8.38 ± 2.66, respectively) and ROM (108.13 ± 16.07) were lower in the fall versus non-fall group.

**Table 2:** Comparison of Falls among Patients of Different Characteristics(N = 372).

Variables	Non-Fallers Group (n = 260)		Fallers Group (n = 112)		χ²/*p*	*t*/*p*
	N (%)	M ± SD	N (%)	M ± SD		
***Patient Characteristics***						
**Age**		68.51 ± 5.23		66.89 ± 4.28		.004*
**BMI**		25.00 ± 3.74		26.60 ± 3.98		<.001**
**Sex**					.15	
Male	40 (78.4)		11 (21.6)			
Female	220 (68.5)		101 (31.5)			
**Legs Affected**					.64	
Unilateral	158(60.7)		73(39.3)			
Bilateral	102(72.3)		39(27.7)			
***Medical History***						
**Pneumonia**					.04	
No	251(69.1)		112(30.9)			
Yes	9(0)		0(0)			
**Diabetes Mellitus**					.00**	
No	241(74.4)		83(25.6)			
Yes	19(39.6)		29(60.4)			
**Neurology Problem (parkinson, stroke**)					.00**	
No	260(71.8)		102(28.2)			
Yes	0(0)		10(100)			
**Heart Disease**					.051	
No	176(73.3)		64(26.7)			
Yes	84(63.6)		48(36.4)			
**Low Back Pain**					.07	
No	147(73.9)		52(26.1)			
Yes	113(65.3)		60(34.7)			
***Knee Functionality***						
**Fear of Falling**		20.22 ± 2.99		30.19 ± 7.93		<.001**
**KOOS**						
Symptom		19.80 ± 2.37		16.85 ± 3.21		<.001**
Pain		19.04 ± 4.86		14.14 ± 3.97		<.001**
Function in activity daily living (ADL)		30.62 ± 7.86		24.17 ± 7.49		<.001**
Function in Sport and Recreation		13.30 ± 5.93		9.51 ± 5.01		<.001**
knee-related Quality of Life		10.09 ± 2.38		8.38 ± 2.66		<.001**
**ROM Knee (degree**)		129.68 ± 11.56		108.13 ± 16.07		<.001**
**Timed Up Go Test (TUG) (second**)		12.73 ± 2.90		16.22 ± 6.07		<.001**
**Proprioception Test**		5.07 ± 3.57		7.25 ± 3.52		<.001**
**Knee Pain (VAS**)		2.50 ± 2.07		4.62 ± 2.25		<.001**
**Chair Stand Test**		9.24 ± 3.45		8.52 ± 4.48		.09

BMI = body mass index, KOOS = knee injury and osteoarthritis outcome source, M = mean, ROM = range of motion, SD = standard deviation, TUG = timed up and go test, VAS = visual analogue scale.

* *P* < .05,

** *P* < .001.

Table 3 summarizes the results of univariate and multivariate logistic regression analyses. Univariate logistic regression showed that age (*P* < .001), BMI (*P* < .001), bilateral knee involvement (*P* = .09), diabetes mellitus (*P* = .01), low back pain (*P* = .02), KOOS symptoms (*P* = .19), KOOS sport and recreation subscales (*P* = .06), knee ROM (*P* < .001), and proprioception test results (*P* < .001) were significantly associated with the risk of falls. Multivariate analysis showed that BMI, diabetes mellitus, low back pain, fear of falls, KOOS symptoms, ROM, and proprioception test results were significantly associated with fall risk. Patients with better proprioception and ROM were less predisposed to falls compared with patients in other groups, (odds ratio [OR] 0.55 and 0.96, respectively). Participants with higher scores on KOOS symptom, fear of falls, low back pain, diabetes mellitus, and high BMI were more likely to fall compared with those from the other groups (OR 1.41, 2.65, 1.27, 3.45, and 1.10, respectively).

**Table 3:** Univariate and Multivariate logistic regression analysis predicting falls among knee Osteoarthritis patients (N = 372).

Variables	Univariate			Multivariate (Backward Selection)		
	OR	95%CI	p value	OR	95%CI	*p* value
***Patient Characteristics***						
**Age**	0.53	0.38–0.74	<0.001**	0.66	0.55–0.80	.06
**BMI**	1.03	0.01–1.23	<0.001**	1.10	0.16–1.56	<.001**
**Sex**						
Male	1.00					
Female	1.08	0.10–2.11	0.31	N/A	N/A	N/A
**Legs Affected**						
Unilateral	1.00					
Bilateral	1.02	0.00–2.06	0.09**	1.09	0.07–1.23	.09
***Medical History***						
**Pneumonia**						
No	1.00					
Yes	0.12	0.00–0.23	0.98	N/A	N/A	N/A
**Diabetes Mellitus**						
No	1.00					
Yes	3.38	1.55–6.20	0.01*	3.45	1.06–5.23	<.001**
**Neurology Problem (parkinson, stroke**)						
No	1.00					
Yes	0.1	0.02–0.24	0.87	N/A	N/A	N/A
**Heart Disease**						
No	1.00					
Yes	1.72	0.11–2.71	0.69	N/A	N/A	N/A
**Low Back Pain**						
No	1.00					
Yes	1.14	0.01–2.76	0.02**	1.27	0.34–3.06	<.001**
***Knee Functionality***						
**Fear of Falling**	12.02	2.40–60.22	0.02*	2.65	1.74–4.01	<.001**
**KOOS**						
Symptom	2.11	0.68–6.45	0.19*	1.41	1.11–1.79	<.001**
Pain	0.78	0.28–2.17	0.64	N/A	N/A	N/A
Function in activity daily living (ADL)	0.57	0.14–2.24	0.42	N/A	N/A	N/A
Function in Sport and Recreation	0.86	0.62–2.75	0.06*	0.94	0.71–2.88	.08
knee-related Quality of Life	1.83	0.55–6.06	0.32	N/A	N/A	N/A
**ROM Knee *(degree***)	0.93	0.46–1.78	<0.001**	0.96	0.76–1.88	<.001**
**Timed Up Go Test (TUG) *(second***)	0.94	0.31–2.92	0.92	N/A	N/A	N/A
**Proprioception Test**	0.19	0.08–0.44	<0.001**	0.55	0.39–0.77	.001**
**Chair Stand Test**	0.38	0.07–1.98	0.25	N/A	N/A	N/A

BMI = body mass index, CI = confidence interval, KOOS = knee injury and osteoarthritis outcome source, OR = odds ratio, ROM = range of motion, TUG = timed up and go test.

* *P* < .05,

** *P* < .001.

## 4. Discussion

In this study, we investigated the main determinants of fall risk in patients with knee OA in Malang, East Java, Indonesia. Our findings show that proprioception and knee ROM can be considered protective factors against falls, whereas KOOS symptoms, fear of falls, low back pain, diabetes mellitus, and elevated BMI tend to be associated with a moderate risk of falls, and diabetes mellitus and fear of falls were shown to be most strongly associated with fall risk. Identification of these predictors is a strength of this study because previous research has not addressed this issue.^[9]^

A study has shown that knee joint proprioception was associated with a fall history among older adults with and without knee OA.^[24]^ The findings of this study are consistent with those reported by previous research^[24,25]^ in which authors observed that improved knee proprioceptive control enhanced single-stance stability and enabled safer interactions with the ground, which consequently reduced fall incidence. Other studies have also reported the importance of proprioception; age-induced loss of proprioceptive ability can alter joint biomechanics and neuromuscular control of the extremities, which results in poor balance and an increased risk of falls.^[26,27]^ Multivariate logistic regression analysis showed that ROM was a key factor associated with falls in patients with knee OA in this study. Knee ROM plays an important role in maintenance of knee function because reduced knee ROM in patients with knee OA secondary to age-induced changes and disease progression was shown to be statistically significant with increased activity limitation and decreased balance, both of which increase fall risk.^[28,29]^ Therefore, specific interventions focused on improved or maintained knee ROM and proprioceptive control are essential in clinical rehabilitation,^[30]^ as recommended by a study in which authors observed better muscle strength, physical function, and ROM in patients with knee OA, who received proprioceptive training than in those who did not receive such training, which can be considered an important contributor to reduced fall risk in elderly patients.

In our current study, 93.75% of patients with OA who experienced falls belonged to the category of patients who were “highly afraid of falls,” and this percentage was lower in the group of patients who had no falls within 1 year prior to the study. This result is consistent with that of a previous study^[31]^ in which 65% of elderly patients who had never had falls experienced fear of falls. This percentage was even higher in elderly individuals who had experienced a fall, with 92% of these individuals having a fear of falls. This condition is also explained by the fear avoidance model theory,^[32]^ which describes how individuals experience chronic musculoskeletal pain as a result of avoidant behavior owing to fear and consequently develop loss of self-confidence and an increasing fear of pain. This condition limits activities and can cause significant muscle atrophy, loss of balance, gait disorder, functional activity limitations, and changes in physical condition, all of which may lead to an increased risk of falls.^[3,10]^

Our findings showed that patients with knee OA and a history of falls had higher KOOS symptom scores than those without previous falls. These results are consistent with those reported by a previous study^[33]^ in which authors observed a higher KOOS symptom score in patients with knee OA, which indicates loss of functional capacity, which leads to reduced lower extremity muscle strength and a high risk of falls. Our findings also showed a higher prevalence of low back pain and diabetes mellitus in patients with knee OA and a history of falls than in those with knee OA without a history of falls. These findings are consistent with those of a previous study in which patients with low back pain (particularly moderate-to-severe pain) had a high risk of falls owing to significant knee pain or poor quadriceps muscle strength.^[13]^ Moreover, patients with chronic diabetes mellitus may have loss of motion associated with sensory impairment and impaired movement coordination and balance, which predispose these patients to falls compared with those without diabetes.^[11]^

Multivariate logistic regression analysis also showed a significantly higher risk of falls (OR 1.10) in patients with knee OA and a high BMI than in those with knee OA without high BMI. Previous studies^[34,35]^ have shown that increasing BMI (>25 kg/m^2^) reduces walking velocity and impairs both static and dynamic stability, which increases the risk of injurious falls in elderly individuals. However, another study^[36]^ reported conflicting results and observed a “U”-shaped association between BMI and frailty; individuals with BMI 25.0 to 29.9 kg/m^2^ were less likely to have frailty compared with individuals of healthy weight or those with obesity. However, BMI is a significant predictor of falls in elderly patients with knee OA.^[37,38]^

Following are the limitations of this study, which should be addressed in future research: Owing to the cross-sectional design, the cause-and-effect association between the fear of falls and actual falls in patients with knee OA who may be predisposed to falls could not be established; Self-reported assessment of study measures serves as a drawback because the findings may be affected by recall bias of fall occurrence and survivor bias. The variables analyzed in this study have not been investigated in previous research,^[9]^ although these were shown to be significant predictors of falls in patients with knee OA.

Therefore, our findings provide strong evidence to policy makers to support the development of fall prevention programs that can be implemented in community health care centers across Indonesia. Such interventions are necessary to deliver individualized, person-centered care and improve fall prevention and care strategies through a systematic process of assessment, intervention, and monitoring aimed at minimizing fall risk.

## 5. Conclusion

This study investigated the predictors of fall risk in patients with mild-to-moderate knee OA. Our findings highlight that knee proprioceptive control and ROM are key factors that protect against falls, whereas KOOS symptoms, fear of falls, low back pain, diabetes mellitus, and BMI serve as risk factors for falls, with diabetes mellitus and fear of falls showing a strong association with falls. Therefore, optimal management of knee OA warrants close attention to fall predictors to establish effective fall prevention programs in Indonesia for patients with knee OA.

## Acknowledgements

The authors acknowledge the work and dedication of Puskesmas Rampal Celaket and Posyandu Lansia Samaan Malang, and all of the participants who kindly took part in the study.

## Author contributions

**Conceptualization:** Cheng-Feng Lin.

**Data curation:** Cheng-Feng Lin.

**Formal analysis:** Cheng-Feng Lin.

**Funding acquisition:** Cheng-Feng Lin.

**Investigation:** Rakhmad Rosadi.

**Methodology:** Rakhmad Rosadi.

**Project administration:** Rakhmad Rosadi.

**Resources:** Rakhmad Rosadi.

**Software:** Amornthaep Jankaew, Li-Chieh Kuo.

**Supervision:** Amornthaep Jankaew, Li-Chieh Kuo.

**Validation:** Amornthaep Jankaew, Po-Ting Wu, Li-Chieh Kuo.

**Visualization:** Amornthaep Jankaew, Po-Ting Wu.

**Writing – original draft:** Rakhmad Rosadi, Po-Ting Wu.

**Writing – review & editing:** Rakhmad Rosadi, Po-Ting Wu.

## Correction

Affiliation a has been corrected to “Institute of Allied Health Sciences, College of Medicine, National Cheng Kung University, Tainan, Taiwan” from “Department of Orthopedics Surgery, College of Medicine, National Cheng Kung University, Tainan, Taiwan.”
